# The Effect of Solar Irradiated *Vibrio cholerae* on the Secretion of Pro-Inflammatory Cytokines and Chemokines by the JAWS II Dendritic Cell Line *In Vitro*

**DOI:** 10.1371/journal.pone.0130190

**Published:** 2015-06-11

**Authors:** Cornelius Cano Ssemakalu, Eunice Ubomba-Jaswa, Keolebogile Shirley Motaung, Michael Pillay

**Affiliations:** 1 Department of Biotechnology, Faculty of Applied and Computer Sciences, Vaal University of Technology, Vanderbijlpark, 1900, Gauteng, South Africa; 2 Council for Scientific and Industrial Research, Natural Resources and the Environment, P.O. Box 395, Pretoria, 0001, Gauteng, South Africa; 3 Department of Biomedical Sciences, Tshwane University of Technology, 175 Nelson Mandela Drive, Arcadia Campus, Pretoria, 0001, Gauteng, South Africa; University of Kansas School of Medicine, UNITED STATES

## Abstract

The use of solar irradiation to sterilize water prior to its consumption has resulted in the reduction of water related illnesses in waterborne disease endemic communities worldwide. Currently, research on solar water disinfection (SODIS) has been directed towards understanding the underlying mechanisms through which solar irradiation inactivates the culturability of microorganisms in water, enhancement of the disinfection process, and the health impact of SODIS water consumption. However, the immunological consequences of SODIS water consumption have not been explored. In this study, we investigated the effect that solar irradiated *V*. *cholerae* may have had on the secretion of cytokines and chemokines by the JAWS II dendritic cell line *in vitro*. The JAWS II dendritic cell line was stimulated with the different strains of *V*. *cholerae* that had been: (i) prepared in PBS, (ii) inactivated through a combination of heat and chemical, (iii) solar irradiated, and (iv) non-solar irradiated, in bottled water. As controls, LPS (1 μg/ml) and CTB (1 μg/ml) were used as stimulants. After 48 hours of stimulation the tissue culture media from each treatment was qualitatively and quantitatively analysed for the presence of IL-1α, IL-1β, IL-6, IL-7, IL-10, IL-12p40, IL-12p70, IL-15, MIP-1α, MIP-1β, MIP-2, RANTES, TNF-α, IL-23 and IL-27. Results showed that solar irradiated cultures of *V*. *cholerae* induced dendritic cells to secrete significant (*p<0*.*05*) levels of pro-inflammatory cytokines in comparison to the unstimulated dendritic cells. Furthermore, the amount of pro-inflammatory cytokines secreted by the dendritic cells in response to solar irradiated cultures of *V*. *cholerae* was not as high as observed in treatments involving non-solar irradiated cultures of *V*. *cholerae* or LPS. Our results suggest that solar irradiated microorganisms are capable of inducing the secretion of pro-inflammatory cytokines and chemokines. This novel finding is key towards understanding the possible immunological consequences of consuming SODIS treated water.

## Introduction

The diseases associated with the consumption of microbiologically contaminated water remains a major global challenge. This is often attributed to poor hygiene and sanitary practices as well as the lack of basic sanitary infrastructure. The World Health Organisation (WHO) and the United Nations International Children's Emergency Fund (UNICEF) in their joint monitoring program on drinking water and sanitation reported that over 700 million people still lacked access to improved sources of clean water and that 1 billion people still practiced open laxation [1]. The practice of open laxation has been associated with the contamination of environmental water sources with faecal material [1,2]. The presence of faecal matter within the natural water resources utilised by the surrounding communities has often resulted in unmanageable water borne disease outbreaks such as typhoid, dysentery and cholera [3,4]. Cholera is a life threatening water borne infection that continues to claim more than 100,000 lives annually [4]. This disease results from infection with pathogenic members of the species of a motile Gram-negative bacterium called *Vibrio cholerae*. *Vibrio cholerae* naturally exists within the aquatic environment [5]. The consumption of untreated environmental water contaminated with *V*. *cholerae* results in the contraction of cholera. The spread of water borne diseases such as cholera could be mitigated through the implementation of preventative measures [6]. These include but are not limited to the provision of basic sanitary infrastructure, treated piped water and proper hygiene and sanitation campaigns. However, the cost associated with the implementation and maintenance of these measures remains a major challenge especially in developing countries [1,6]. The plight of communities worldwide suffering from infections acquired through the consumption of water borne pathogens could be addressed through the use of solar disinfection (SODIS) of water in conjunction with currently available preventative and crisis control measures. SODIS of water could be regarded as an ideal intervention because it is easy to use, sustainable and compatible to the life style encountered in resource poor communities [6].

The consumption of SODIS water has been shown to curb the number of water borne disease infections especially in resource poor countries within the Sub Saharan African, East Asian and South American regions [7–9]. SODIS relies on Solar Ultra Violet Radiation (SUVR) to render microbiologically contaminated water safe for consumption. SUVR inactivates the culturability of various microorganisms [10,11] through the formation of photosensitisers through photo-oxidation [12–14]. Photosensitizers damage the cell membrane [15,16], induce irreversible damage to the catalyse system [17,18] and block ATP synthesis [19,20]. This shows that the methods through which the photosensitisers inactivate the microorganisms is ambiguous and hence there is great variability in microbial states following SUVR.

To date, research has focused on studies pertaining to the health impact of SODIS interventions [7,8,21], enhancement of the SODIS technology [14,22] and mechanisms of SODIS inactivation of microorganisms [16,19]. However, the immunological connotations pertaining to the consumption of SODIS water have not been explored. It is possible that the epidemiological benefits observed in SODIS water consumers could also be as a result of the immunological benefits [6].

In this study, we investigated the effect that solar irradiated *V*. *cholerae* may have had on the profile of cytokines and chemokines secreted by dendritic cells *in vitro*. Dendritic cells are versatile professional antigen presenting cells found in peripheral tissues, lymph and secondary organs as well as regions where macrophages and B-cells are generally excluded. The dendritic cells’ versatility lies in their ability to bridge the gap between the innate and adaptive immune response as well as modulate the type of adaptive immune response [23]. Dendritic cells are able to modulate the type of immune response through the cascade of cytokines and chemokines they produce during their encounter with thymus lymphocytes (T-cells). Depending on the nature of the antigens and level of maturation, the dendritic cells through the cascade of cytokines and chemokines they secrete may induce protective T helper (Th) 1 or Th2 cell responses or T regulatory cells [24]. Dendritic cells secrete a variety of cytokines as well as chemokines in response to microbial invasion as well as their by-products [25]. Cytokines that induce inflammation such as interleukin (IL)-1, IL-6, IL-8, IL-12, IL-18 and Tumor Necrosis Factor (TNF)-α are referred to as pro-inflammatory cytokines [26]. Cytokines such as IL-4, IL-10, IL-13 and Transforming Growth Factor (TGF)-β that inhibit the inflammatory processes are called anti-inflammatory cytokines [25]. The secretion of pro-inflammatory cytokines is desirable during an active infection. However, dysregulation of pro-inflammatory cytokine secretion results in gross tissue damage and apoptosis [27]. The cascade of cytokines and chemokines secreted in response to a microorganism relies on the state of the microbial cell or microbial components [24,28–30] which also depends on the conditions under which the microorganism existed prior to its consumption. Microbial cells in nature, especially those exposed to stressors, exhibit substantial variability in their cell membrane [15] and protein integrity [31]. Furthermore, solar irradiated bacterial cells have been shown to have a different protein profile when compared to the non-solar irradiated bacterial cells [20]. It was therefore important to understand the nature and extent of cytokines and chemokines secreted by the dendritic cells in response to solar irradiated bacterial cells.

In this study an immortalized dendritic cell line known as JAWS II that was established from the bone marrow cultures of p53 ^-/-^ C57BL/6 mice [32] was used. This cell line has been used in antitumor and pathogen specific immunity studies [30]. Furthermore, it has also been shown to display similar maturation characteristics such as those exhibited by bone marrow derived dendritic cells (BMDCs) [30,33]. The use of this cell line, as opposed to murine BMDCs, negates the need to slaughter numerous mice for their bone marrow and ensures reproducible results that could be interpreted easily [30].

## Materials and Methods

### Cell culture reagents

Iscove’s Modified Dulbecco’s Medium (IMDM), and sterile 1x Phosphate Buffer Saline (PBS) were purchased from Life technologies (Carlsbad, CA); antibiotics penicillin and streptomycin were purchased from BioWhittaker (Walkersville, MD) whereas gentamicin was purchased from Melford (Chelsworth, United Kingdom); Granulocyte Macrophage-Colony Stimulating Factor (GM-CSF) was purchased from Abcam (Cambridge, United Kingdom); Fetal Bovine Serum (FBS) was purchased from Thermo scientific (Waltham, MA); the rough form lipopolysaccharide (LPS) from *E*. *coli* serotype J5 and the cholera toxin beta-subunit (CTB) were purchased from ENZO Life Sciences (Farmingdale, NY); 2-mercaptoethanol (2-ME) and 0.25% Trypsin-0.02% EDTA were purchased from Sigma (St. Louis, MO).

### JAWS II Dendritic Cell Culture

An immortalised dendritic cell line established from the bone marrow of a p53-knockout C57BL/6 mouse was used for this study [32]. This GM-CSF dependent dendritic cell line also known as the JAWS II cell line was obtained from the American Type Culture Collection (CRL-11904; ATCC Manassas, VA). Cells were grown in a Carbon Dioxide (CO_2_) incubator (Thermo Scientific), at 37°C with 5% CO_2_ in complete culture medium consisting of IMDM supplemented with 10% FBS, 10 U/ml penicillin and 100 μg/ml streptomycin, 0.5 mM 2-ME and 5 ng/ml murine GM-CSF. The medium was pre-incubated at 37°C for at least 15 min to allow it to reach the desirable pH (7.0 to 7.6) prior to the addition of the cells. The cell cultures were maintained by transferring the non-adherent cells into a centrifuge tube. The remaining adherent cells were washed with 1x PBS to remove any traces of FBS and then treated with a solution consisting of 0.25% trypsin -0.02% EDTA at 37°C for 2 min. The two cell suspensions were centrifuged together at 1000 rpm for 10 min in a single centrifuge tube, and the supernatant was discarded. The cell pellet was washed with 1x PBS and resuspended in fresh complete culture medium.

### *Vibrio cholerae* strains

Three previously characterised strains of *V*. *cholerae*—two toxigenic and one non-toxigenic—were used in this study: G4222 was isolated from a cholera patient in Gauteng, South Africa; BRITS01 was isolated from Brits, South Africa and ENV1009 was isolated at Rand Water (Vereeniging) in South Africa [34]. These strains were chosen based on their ability to produce and secrete the cholera toxin [34].

### Stimulation of the JAWS II Dendritic cell line with *V*. *cholerae*

The established JAWS II dendritic cell line was stimulated with the different strains of *V*. *cholerae* that had been: (i) prepared in PBS, (ii) inactivated through a combination of heat and chemical means [35], (iii) solar irradiated and (iv) non-solar irradiated, in bottled water. Bacterial cultures that had either been solar irradiated for 7 hours or inactivated through a combination of heat and chemical means, were no longer culturable on Luria agar. However, the bacterial cultures that were non-solar irradiated as well as those prepared in PBS remained culturable prior to their use in the dendritic cell stimulation experiments. The dendritic cells were co-incubated with *V*. *cholerae* at a multiplicity of infection of 10. As controls, LPS (1 μg/ml), CTB (1 μg/ml) and PBS were used as stimulants. Stimulation of the JAWS II dendritic cell line was done in complete growth medium without antibiotics and incubated at 37°C in a 5% CO_2_ humidified incubator. Antibiotics including gentamicin at 100 μg/ml were added after 4 hours of stimulation and the experiments were incubated further for another 44 hours.

### Sample preparation prior to cytokine and chemokine analysis

After the stimulation experiments, samples were centrifuged at 1000 rpm for 10 min. Then the serum fraction for each sample was transferred to a new tube, which was further centrifuged at 3000 rpm for 5 min to remove any residual cells or debris. The serum was thereafter aliquoted in small single-use volumes to prevent multiple freeze-thaw cycles. The samples were then stored at -20°C until they were required.

### Cytokine and chemokine detection

Cytokines were measured using a Bio-Plex 200 system (Bio-Rad, Hercules, CA) and BioManager software (Bio-Rad, Hercules, CA) for analysis. The following multiplex kits were purchased from Merck Millipore (Billerica, MA): (i) the MCYTOMAG-70K Mouse Cytokine/Chemokine kit, containing IL-1α, IL-1β, IL-6, IL-7, IL-10, IL-12p40, IL-12p70, IL-15, macrophage inflammatory protein 1α (MIP-1α), MIP-1β, MIP-2, RANTES and tumor necrosis factor alpha (TNF-α); (ii) the MCYP3MAG-74K Mouse Cytokine/Chemokine kit, containing IL-23 and IL-27. The kits were run according to the manufacturer’s instructions, with the exception of sample collection and processing as described in the previous subsection. The samples were incubated for 2 hours at room temperature (25°C). All the samples were run on a single plate so as to avoid plate to plate variability.

### Statistical analysis

Statistical analysis was conducted using a student’s T test. Differences at the *P* < 0.05 level were considered statistically significant. The data are expressed as means ± standard error of the mean (SEM) obtained from three biological replicates for each experiment.

## Results

### Dendritic cells stimulated with Solar Irradiated *V*. *cholerae* produced low levels of Il-6

Following 48 hours of dendritic cell stimulation, the production of IL-6 was measured. The unstimulated dendritic cells produced 7.3 ± 4.78 pg/ml of IL-6. However, dendritic cells stimulated with LPS and CTB secreted 3780.97 ± 256.7 pg/ml and 104.8 ± 49.31 pg/ml of IL-6, respectively. When the dendritic cells were stimulated with the differently treated cultures of *V*. *cholerae*, a difference in the expression of IL-6 was observed (Table 1). Non-solar irradiated cultures of *V*. *cholerae* induced the secretion of the greatest amount of IL-6.

**Table 1:** Concentration (Mean ± SEM) of IL-6 expressed by dendritic cells following 48 hours of stimulation with *V*. *cholerae*, LPS and CTB.

*V*. *cholerae* Strain	Mode of *V*. *cholerae* treatment			
	Solar Irradiated (pg/ml)	Non-solar Irradiated (pg/ml)	Prepared in PBS(pg/ml)	Heat/Chemical Inactivation (pg/ml)
**G4222**	31.10 ± 1.09*	12338.10 ± 23.78*	8.01 ± 4.42	**BDL
**BRITS01**	37.54 ± 2.70*	11274.23 ± 38.55*	987.35 ± 425.85	**BDL
**ENV1009**	108.86 ± 4.48*	10814.12 ± 144.98*	56.95 ± 29.76	**BDL
	**Other dendritic cell treatments**			
	**Unstimulated (pg/ml)**	**LPS (pg/ml)**	**CTB (pg/ml)**	
	7.3 ± 4.78	3780.97 ± 256.7*	104.8 ± 49.31	

* Indicates means that had a p< 0.05 in comparison to the untreated dendritic cells.

Heat/Chemical inactivation refers to *V*. *cholerae* cultures that were treated with a combination of heat (65°C) and 1.5% formalin.

**BDL (below the detection limit (< 3.2 pg/ml)).

Unstimulated refers to dendritic cells treated with PBS only.

Solar irradiated cultures of *V*. *cholerae* also induced the expression of IL-6 but it was not as high as that observed when non-solar irradiated cultures of *V*. *cholerae*, LPS and CTB were used as stimulants. However, there was a significant (p<0.05) difference in the means of the IL-6 expression between the dendritic cells which had been stimulated with solar irradiated cultures of *V*. *cholerae* and the unstimulated dendritic cells. Heat and chemically inactivated *V*. *cholerae* cultures did not induce any detectable amount of IL-6.

### Expression of Chemokines MIP-1α, MIP-1β, MIP-2 and RANTES

The expression of chemokines MIP-1α, MIP-1β, MIP-2 and RANTES by the dendritic cells following 48 hours of stimulation was assessed. When the dendritic cells were stimulated with LPS there was a significant (p<0.05) increase in the secretion of chemokines MIP-1α, MIP-1β, MIP-2 and RANTES when compared to that observed in the unstimulated dendritic cells (Table 2). CTB treated dendritic cells on the other hand only expressed MIP-2 whereas MIP-1α, MIP-1β and RANTES were below the detection limit (Table 2).

**Table 2:** Concentration (Mean ± SEM) of chemokines MIP-1α, MIP-1β, MIP-2 and RANTES by dendritic cells following 48 hours of stimulation with LPS and CTB.

	MIP-1α (pg/ml)	MIP-1β (pg/ml)	MIP-2 (pg/ml)	RANTES (pg/ml)
**Unstimulated**	91.73 ± 16.65	BDL	230.87 ± 48.68	BDL
**LPS**	5424.72 ± 707.41*	325.28 ± 53.32	**ADL	83.40 ± 3.82
**CTB**	BDL	BDL	391.44 ± 74.24	***BDL

* Indicates means that had a p< 0.05 in comparison to the untreated dendritic cells.

**ADL (above the detection limit (> 10000 pg/ml)).

***BDL (below the detection limit (< 3.2 pg/ml)).

Unstimulated refers to dendritic cells treated with PBS only.

When the dendritic cells were stimulated with cultures of *V*. *cholerae* that had either been solar irradiated or non-solar irradiated in water, a significant (p<0.05) difference in means of expression of chemokines MIP-1α and MIP-2 in comparison to the unstimulated cells was observed (Table 3).

**Table 3:** Concentration (Mean ± SEM) of chemokines MIP-1α, MIP-1β, MIP-2 and RANTES by dendritic cells following 48 hours of stimulation with *V*. *cholerae*.

Chemokine		Mode of *V*. *cholerae* treatment			
	*V*. *cholerae* Strain	Solar Irradiated (pg/ml)	Non-solar Irradiated (pg/ml)	Prepared in PBS (pg/ml)	Heat/Chemical Inactivation (pg/ml)
**MIP-1α**	G4222	281.46 ± 23.81 *	6622.36 ± 203.86*	64.27 ± 10.96	BDL
	BRITS01	209.65 ± 10.22*	7368.91 ± 343.03*	1883.72 ± 777.13	43.09 ± 28.79
	ENV1009	516.18 ± 23.15*	9696.51 ± 1194.68*	250.53 ± 128.20	43.88 ± 7.6
**MIP-1β**	G4222	17.81 ± 11.39	502.31 ± 20.62	BDL	BDL
	BRITS01	BDL	1473.82 ± 179.85	53.77 ± 33.98	BDL
	ENV1009	41.89 ± 20.67	1647.45 ± 25.11	BDL	BDL
**MIP-2**	G4222	2931.27 ± 244.51*	ADL	543.25 ± 56.63*	BDL
	BRITS01	2295.56 ± 34.68*	ADL	2166.04 ± 1357.96	15.18 ± 8.98
	ENV1009	3082.85 ± 19.82*	ADL	1362.22 ± 249.54*	54.07 ± 20.63
**RANTES**	G4222	3.45 ± 0.08	153.32 ± 8.77	BDL	BDL
	BRITS01	3.06 ± 0.12	641.12 ± 26.4	10.73 ± 3.94	BDL
	ENV1009	3.42 ± 0.1	827.04 ± 64.87	BDL	0.8 ± 0.48

* Indicates means that had a p< 0.05 in comparison to the untreated dendritic cells.

Heat/Chemical inactivation refers to *V*. *cholerae* cultures that were treated with a combination of heat (65°C) and 1.5% formalin. ADL indicates a concentration that was above the detection limit. The highest detection value for this assay was 10000 pg/ml. BDL indicates s concentration that was below the detection limit. The lowest detection value for the assay was 3.2 pg/ml.

However, dendritic cells stimulated with the non-solar irradiated cultures of *V*. *cholerae* expressed higher concentrations of all four chemokines in comparison to the dendritic cells that had been stimulated with solar irradiated cultures of *V*. *cholerae* and LPS (Tables 2 and 3). When PBS prepared cultures of *V*. *cholerae* were used as stimulants there was a higher secretion of chemokines MIP-1α and MIP-2 in comparison to that produced by the unstimulated dendritic cells. However, the secretion of MIP-1β and RANTES was only observed in dendritic cells that had been stimulated with the PBS prepared BRITS01 strain of *V*. *cholerae*. The other two strains of *V*. *cholerae* (G4222 and ENV1009) that had been prepared in PBS did not induce the secretion of detectable amounts of MIP-1β and RANTES by dendritic cells. Heat and chemically inactivated *V*. *cholerae* failed to induce the secretion of detectable amounts of MIP-1β by dendritic cells. Heat/chemical inactivated strains BRITS01 and ENV1009 of *V*. *cholerae* induced very low levels of expression of MIP-1 α and MIP-2 in dendritic cells. Of the heat/chemically inactivated strains of *V*. *cholerae* only the ENV1009 strain was capable of inducing the expression of RANTES (Table 3).

### Dendritic cells stimulated with non-solar Irradiated *V*. *cholerae* produced IL-15

**Fig 1:**
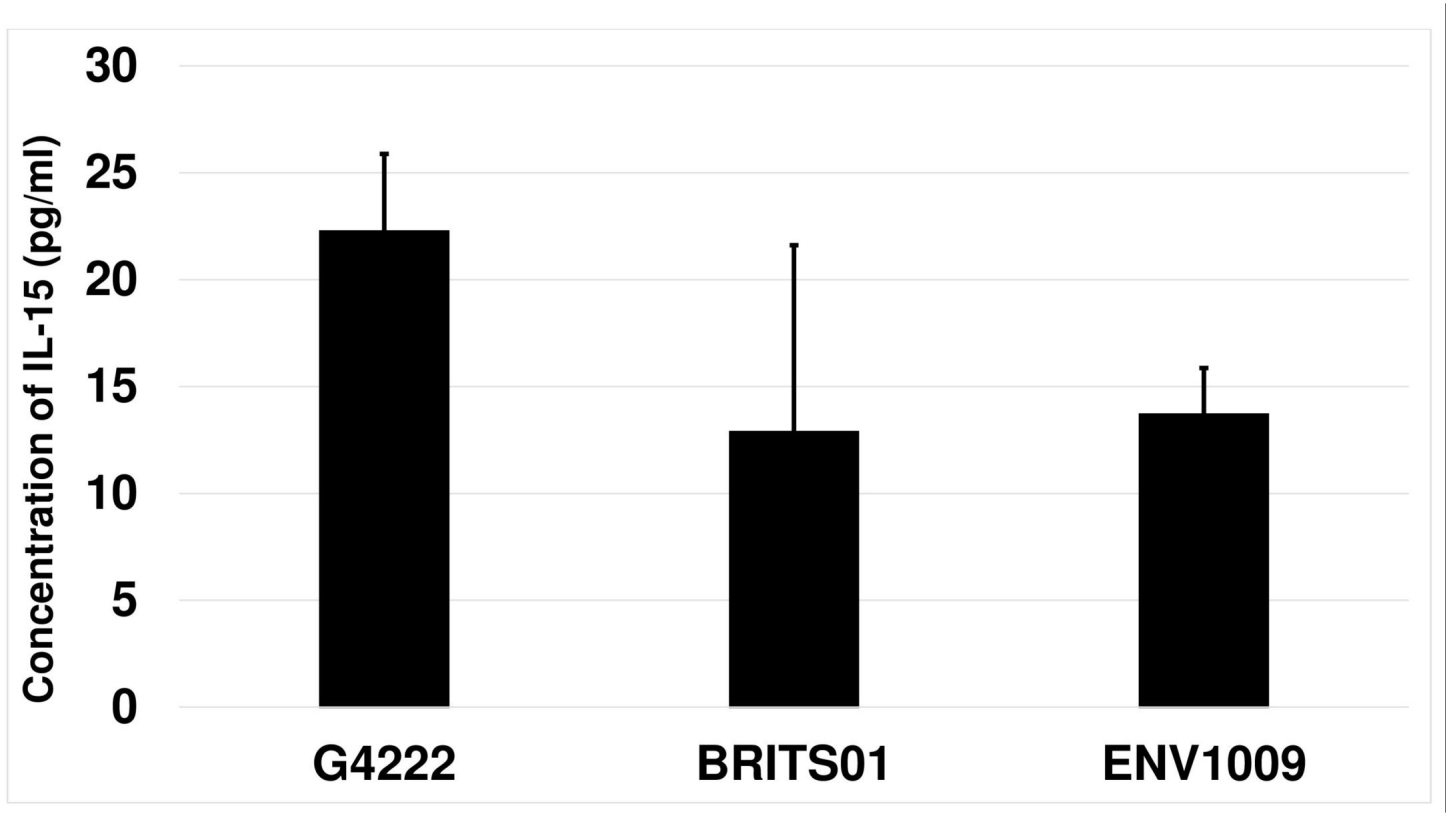
Secretions of IL-15 by dendritic cells. Following 48 hours of stimulation, the amount of IL-23 produced by the dendritic cells stimulated with non-solar irradiated strains G4222, BRITS01 and ENV1009 of *V*. *cholerae* was measured. The error bars indicate the mean standard error of three independent experiments.

The expression of IL-15 by dendritic cells after the 48 hours of stimulation was assessed. The unstimulated dendritic cells as well as those stimulated with LPS and CTB did not secrete any detectable amounts of IL-15 (detection limit was 3.2 pg/ml). A similar result was observed when the dendritic cells were stimulated with solar irradiated, PBS prepared and heat/chemically inactivated cultures of *V*. *cholerae*. Only non-solar irradiated cultures of *V*. *cholerae* induced the secretion of detectable amounts of IL-15 (Fig 1).

### Dendritic cells stimulated with Solar Irradiated *V*. *cholerae* produced low levels of TNF-α

After 48 hours of incubation there was no detectable amounts of TNF-α produced by the unstimulated dendritic cells. A similar result was observed when the dendritic cells were stimulated with CTB as well as heat/chemically inactivated cultures of *V*. *cholerae*. However, LPS induced the expression of TNF-α (197.91 ± 14.79 pg/ml) in dendritic cells. When PBS prepared, solar irradiated and non-solar irradiated cultures of *V*. *cholerae* were used as stimulants, a detectable amount of TNF-α was secreted by the dendritic cells (Table 4).

**Table 4:** Concentration (Mean ± SEM) of TNF-α by dendritic cells following 48 hours of stimulation with *V*. *cholerae*, LPS and CTB.

*V*. *cholerae* Strain	Mode of *V*. *cholerae* treatment			
	Solar Irradiated (pg/ml)	Non-solar Irradiated (pg/ml)	PBS Prepared(Pg/ml)	*Heat/Chemical Inactivation (pg/ml)
**G4222**	6.4 ± 0.44	382.99 ± 13.43	4.54 ± 2.6	**BDL
**BRITS01**	19.86 ± 3.63	641.86 ± 55.31	89.54 ± 31.54	**BDL
**ENV1009**	18.31 ± 1.88	961 ± 29.28	22.83 ± 9.33	**BDL
	**Other dendritic cell treatments**			
	**Unstimulated (pg/ml)**	**LPS (pg/ml)**	**CTB (pg/ml)**	
	**BDL	197.91 ± 14.79	**BDL	

*Heat/Chemical inactivation refers to *V*. *cholerae* cultures that were treated with a combination of heat (65°C) and 1.5% formalin.

** BDL (below the detection limit (< 3.2 pg/ml)).

Unstimulated refers to dendritic cells treated with PBS only.

The non-solar irradiated cultures of *V*. *cholerae* in water induced the secretion of the highest amounts of TNF-α in comparison to all the other treatments while the solar irradiated cultures of *V*. *cholerae* in water resulted in the lowest expression of TNF-α (Table 4).

### Expression of cytokines IL-1α and IL-1β

The expression of cytokines IL-1α and IL-1β by the dendritic cells following 48 hours of stimulation was assessed. When the dendritic cells were stimulated with LPS there was a significant increase in the secretion of IL-1α but not IL-1β (Table 5).

**Table 5:** Concentration (Mean ± SEM) of cytokines IL-1α and IL-1β by dendritic cells following 48 hours of stimulation with *V*. *cholerae*, LPS and CTB.

Chemokine		Mode of *V*. *cholerae* treatment			
	*V*. *cholerae* Strain	Solar Irradiated (pg/ml)	Non-solar Irradiated (pg/ml)	PBS Prepared (pg/ml)	Heat/Chemical Inactivation (pg/ml)
**IL-1α**	G4222	119.31 ± 1.61*	3849.91 ± 386.20*	44.17 ± 27.72	BDL
	BRITS01	131.9 ± 3.74*	2568.32 ± 96.73*	321.63 ± 88.89*	BDL
	ENV1009	170.96 ± 2.91*	3317.77 ± 138.58*	76.37 ± 41.32	5.19 ± 3.66
**IL-1β**	G4222	1.99 ± 1.16	133.11 ± 3.55	BDL	BDL
	BRITS01	BDL	BDL	BDL	BDL
	ENV1009	BDL	71.85 ± 4.91	BDL	BDL
		**Other dendritic cell treatments**			
		**Unstimulated (pg/ml)**	**LPS (pg/ml)**	**CTB (pg/ml)**	
	**IL-1α**	37.79 ± 3.55	783.91 ± 44.63*	23.94 ± 6.55	
	**IL-1β**	BDL	BDL	BDL	

* Indicates means that had a p< 0.05 in comparison to the untreated dendritic cells.

Heat/Chemical inactivation refers to *V*. *cholerae* cultures that were treated with a combination of heat (65°C) and 1.5% formalin. ADL indicates a concentration that was above the detection limit. The highest detection value for this assay was 10000 pg/ml. BDL indicates s concentration that was below the detection limit. The lowest detection value for the assay was 3.2 pg/ml. Unstimulated refers to dendritic cells treated with PBS only.

LPS did not induce the dendritic cells to produce any detectable amounts of IL-1β. CTB on the other hand induced the dendritic cells to express 23.94 ± 6.55 pg/ml of IL-1α. However, the amount of IL-1α produced by CTB stimulated dendritic cells was lower than that observed in the unstimulated dendritic cells (37.79 ± 3.55 pg/ml). CTB did not induce the secretion of IL-1β by the dendritic cells. When the dendritic cells were stimulated with cultures of *V*. *cholerae* and assessed for the secretion of IL-1α and IL-1β, an increase in the secretion of IL-1α was observed (Table 5). However the secretion of IL-1β was not observed when heat/chemically inactivated as well as PBS prepared cultures of *V*. *cholerae* were used as stimulants. The non-solar irradiated cultures of *V*. *cholerae* induced dendritic cells to secrete the highest amounts of IL-1α in comparison to the levels observed in unstimulated dendritic cells (Table 5).

### Dendritic cells stimulated with solar Irradiated *V*. *cholerae* produced IL-23

Following the 48 hours incubation, there was no detectable amounts of IL-23 produced by the unstimulated dendritic cells. A similar result was observed when the dendritic cells were stimulated with LPS and CTB. But when the dendritic cells were stimulated with the *V*. *cholerae* cultures, only the solar irradiated strains of *V*. *cholerae* induced the secretion of IL-23 (Table 6).

**Table 6:** Concentration (Mean ± SEM) of IL-23 by dendritic cells following 48 hours of stimulation with *V*. *cholerae*.

*V*. *cholerae* Strain	Mode of *V*. *cholerae* treatment			
	Solar Irradiated (pg/ml)	Non-solar Irradiated (pg/ml)	PBS Prepared (pg/ml)	Heat/Chemical Inactivation (pg/ml)
**G4222**	7.35 ± 4.42	BDL	10.19 ± 7.09	BDL
**BRITS01**	4.51 ± 2.25	4.51 ± 2.25	BDL	BDL
**ENV1009**	10.19 ± 7.09	BDL	BDL	24.4 ± 12.45

Heat/Chemical inactivation refers to *V*. *cholerae* cultures that were treated with a combination of heat (65°C) and 1.5% formalin. BDL indicates a concentration that was below the detection limit. The lowest detection value for this assay was 3.2 pg/ml.

However, the ability of the other modes of *V*. *cholerae* treatments (PBS prepared, non-solar irradiated, and heat/chemically inactivated) to induce IL-23 secretion by the dendritic cells was strain specific. Of all the PBS prepared cultures of *V*. *cholerae* only the culture consisting of the G4222 strain was capable of inducing dendritic cells to secrete IL-23. Similarly, of all the heat/chemically inactivated cultures of *V*. *cholerae* only the culture consisting of the ENV1009 strain was capable of inducing dendritic cells to secrete IL-23 (Table 6). Of all the non-solar irradiated cultures of *V*. *cholerae* only the culture consisting of the BRITS01 strain was capable of inducing dendritic cells to secrete IL-23.

### Cytokines below detection limit

Cytokines IL-7, IL-10, IL-12p40, IL-12p70 and IL-27 were below the detection limit following dendritic cell stimulation.

## Discussion

In this study, we investigated the profiles of cytokines and chemokines secreted by dendritic cells *in vitro* following their stimulation with differently prepared cultures of *V*. *cholerae*. Microorganisms as well as their cellular products have been shown to induce the secretion of cytokines and chemokines by dendritic cells *in vivo* and *in vitro*. Cytokines are low molecular weight peptides, proteins or glycoproteins produced by nucleated cells [26,36] and are essential mediators of an immunologic response [37]. Chemokines are a subset of cytokines whose primary role is to attract cells of the immune system to the site of infection as well as regulate their activities [38].

IL-6 is a pleiotropic pro-inflammatory cytokine that has been shown to enhance the survival of naïve T-cells, and proliferation of B-cells and CD8+ T-cells [39]. However, sustained secretion of IL-6 may not be desirable as it has been shown to play a role in chronic inflammatory proliferation especially that resulting from connective tissue breakdown [40]. The results of this study showed that the non-solar irradiated cultures of *V*. *cholerae* induced the dendritic cells to secrete the highest levels of IL-6 (Table 1) as opposed to other treatments. Furthermore, the dendritic cells stimulated with the non-solar irradiated toxigenic strains of *V*. *cholerae* (G4222 and BRITS01) produced more IL-6 than the non-toxigenic non-solar irradiated environmental strain (ENV1009). The difference between these strains was in their ability to produce the cholera toxin. Strains G4222 and BRITS01 unlike the ENV1009 strain of *V*. *cholerae* have been shown to produce the cholera toxin [34]. This observation could be explained by the ability of the toxigenic strains to produce the cholera toxin. The cholera toxin in its entirety has been reported to induce strong production of IL-6 [29] while the use of only the beta subunit of the cholera toxin results in suppressed IL-6 production [41]. When the dendritic cells were stimulated with solar irradiated cultures of *V*. *cholerae*, a significant increase in the secretion of IL-6 was observed at *a p<0*.*05* level vs the unstimulated dendritic cells (Table 1). Although significant, the magnitude of IL-6 secreted by dendritic cells in response to solar irradiated cultures of *V*. *cholerae* was not as high as that observed when LPS, CTB and non-solar irradiated cultures of *V*. *cholerae* were used as stimulants. This finding suggests that solar irradiated cultures of *V*. *cholerae* were able to induce a significant increase in the secretion of IL-6. However, it remains unclear if the dose of IL-6 secreted by the dendritic cells in response to the solar irradiated cultures is immunologically meaningful.

TNF-α is a pro-inflammatory cytokine that induces the secretion of other cytokines including itself [42] and signals for the destruction of bacterial cells through the activation of pathways producing nitric oxide. LPS a known inducer of pro-inflammatory secretion induced the dendritic cells to secrete TNF-α (Table 4). However, the concentration of TNF-α produced by the dendritic cells in response to LPS (197.91 ± 14.79 pg/ml) was not as high as that observed in stimulations involving the non-solar irradiated cultures of *V*. *cholerae* (Table 4). This observation could be due to presence of more LPS molecules on the intact bacterial cells as opposed to the concentration of the purified LPS used during the dendritic cell stimulation [43]. Dendritic cells stimulated with CTB did not secrete any detectable amounts of TNF-α. This finding confirms observations made by Burkart et al. [41] as well as Karla and Satchell [44] regarding the effect of the cholera toxin on the secretion of TNF-α. These researchers found that the cholera toxin down regulated the synthesis of TNF-α thereby inhibiting the innate immunity at the earliest steps of infection. We also found that heat/chemically inactivated cultures of *V*. *cholerae*, just like the CTB did not induce the dendritic cells to secrete TNF-α. On the other hand solar irradiated cultures of *V*. *cholerae* induced the dendritic cells to secrete minimal amounts of TNF-α *in vitro* (Table 4). However, the concentration of TNF-α produced by the dendritic cells stimulated with solar irradiated cultures of *V*. *cholerae* was not as high as that observed when LPS, non-solar irradiated cultures and PBS prepared cultures of *V*. *cholerae* were used as stimulants (Table 4).

IL-1 is a pro-inflammatory cytokine that is produced in two forms, namely, IL-1α and IL-1β [36]. Although these forms may appear to be structurally different they have been reported to act through the same receptors [36] with IL-1β being the most potent [40]. IL-1 being a highly pro-inflammatory cytokine, its production plays a pivotal role between clinical benefits and unacceptable toxicity [40]. The presence of high concentrations of IL-1 has been shown to result in fever, tissue destruction, and in some cases shock and death [26]. The presence of IL-1β has been reported to aid in the replication of the Human immunodeficiency virus (HIV) through the induction of apoptosis in both the naïve CD4+ and CD8+ T- cells [37, 45,46]. LPS has been reported to trigger an inflammatory response characterised by the production of IL-1 among other pro-inflammatory cytokines [47]. In this study the concentration of IL-1α produced by the dendritic cells in response to LPS was not as high as that observed when the non-solar irradiated cultures of *V*. *cholerae* were used. The consumption of non-solar irradiated strains of *V*. *cholerae* in water may induce the secretion of high concentrations of pro-inflammatory cytokine [29,48]. Our results showed that solar irradiated cultures of *V*. *cholerae* induced a significant increase in the expression of IL-1α by the dendritic cells (Table 5). The ability for solar irradiated cultures of *V*. *cholerae* to induce the secretion of a significant amount (as compared to the control) of IL-1α that was lower than that observed when LPS or non-solar irradiated cultures of *V*. *cholerae* were used as stimulant may be beneficial to the immune system of the consumer. However, it is probably worth noting that the increase in the concentration of IL-1α secreted by the dendritic cells stimulated with solar irradiated *V*. *cholerae* was not as high as that observed when LPS or non-solar irradiated cultures of *V*. *cholerae* were used as stimulants (Table 5). Treatments involving non-solar irradiated strains (G4222 and ENV1009) induced a strong secretion of IL-1β by the dendritic cells (Table 5). All the other treatments did not induce the secretion of IL-1β above the detection limit.

Besides IL-6, TNF-α and IL-1, solar irradiated cultures of *V*. *cholerae* were shown to induce the expression of a minimal amount of IL-23 (Table 6). IL-23 is an IL-12 family cytokine composed of IL-12p40 subunit and a novel cytokine subunit p19. IL-23 has been reported to play a significant role in infectious diseases, autoimmunity, and cancer, marked by its ability to promote IL-17 secreting T-cells [49]. The ability for all the solar irradiated cultures of *V*. *cholerae* to induce the secretion of IL-23 cytokine by the dendritic cells unlike other treatments suggests that solar irradiation of the *V*. *cholerae* strains may have led to the availability of microbial cellular components that are key to the secretion of IL-23. Microbial cellular components such as those that make up outer membrane vesicles, have been reported to induce the secretion of IL-23 [50,51]. *Vibrio cholerae* contains most of these microbial components [50] but solar irradiation could have resulted in their availability. The secretion of IL-23 by the dendritic cells following their stimulation with non-solar irradiated, PBS prepared or heat/chemically inactivated cultures of *V*. *cholerae* was strain specific (Table 6).

Apart from the cytokines, the solar irradiated cultures of *V*. *cholerae* were investigated for their ability to induce the secretion of chemokines MIP-1α, MIP-1β, MIP-2 and RANTES. MIP-2 was the most produced chemokine by the dendritic cells following their stimulation with solar irradiated cultures of *V*. *cholerae*. MIP-2 is a chemotactic chemokine that induces localised neutrophil infiltration. Neutrophils have been shown to retard the course of cholera infection by restricting the microorganisms to the intestines. The absence of neutrophils results in the spread of the microorganisms to extra-intestinal organs such as the spleen which could result in a systemic increase of IL-1β and TNF-α [52]. MIP-2 has also been reported to attract monocytes and T-cell receptor-αβ positive cells circulating in blood [53,54]. LPS and the non-solar irradiated cultures of *V*. *cholerae* induced dendritic cells to produce over 10,000pg/ml of MIP-2 (Table 3). The production of a high concentration MIP-2 may not be beneficial to the host as it has been linked to gross tissue damage in corneal disease [54] as well as acute pancreatitis [55]. The concentration of MIP-2 produced by the dendritic cells stimulated with solar irradiated cultures of *V*. *cholerae* in this study was not as high as that observed in studies reporting gross tissue damage (Table 3). The solar irradiated cultures of *V*. *cholerae* also induced the dendritic cells to secrete a significant amount of MIP-1α. MIP-1α has been reported to play a critical role in Th1 polarisation through its ability to up regulate the expression of B7-1 and B7-2 receptors on antigen presenting cells [38]. However, just like MIP-2, the dendritic cells secreted a higher concentration of MIP-1α when LPS and the non-solar irradiated cultures of *V*. *cholerae* were used as stimulants (Table 3). High concentrations of MIP-1α such as those observed this study when LPS and non-solar irradiated cultures of *V*. *cholerae* were used as dendritic cell stimulants could result in tissue damage such as that observed in multiple myeloma [56,57]. The secretion of MIP-1β and RANTES following the stimulation of the dendritic cells with all the different stimulants was not as pronounced as in the case of MIP-1α or MIP-2. MIP-1β and RANTES are inflammatory chemokines that primarily target monocytes and Th1 T-cells. LPS has been reported to induce dendritic cells to secrete MIP-1β [58]. In this study, the concentration of MIP-1β secreted by the dendritic cells resulting from their stimulation with LPS was lower than that observed when non-solar irradiated cultures of *V*. *cholerae* were used as stimulants. A similar result was noticed in regard to RANTES. This finding seems to suggest that LPS could not have been the only stimulant of MIP-1β and RANTES. It is possible that the viable cultures of *V*. *cholerae* in the water environment unlike those in PBS could have produced compounds with the ability to induce the secretion of high levels of MIP-1β and RANTES by the dendritic cells.

These observations indicated that the mode of microbial inactivation influences the nature and magnitude of the cytokine as well as chemokines secreted. When non-solar irradiated and PBS prepared cultures of *V*. *cholerae* were used to stimulate dendritic cells a difference in cytokine and chemokine response was observed. Both the non-solar irradiated and PBS prepared cultures of *V*. *cholerae* contained viable bacterial cells. The only difference in these cultures was the medium in which they were prepared. In addition, *V*. *cholerae* cultures that were heat and chemically inactivated as well as solar irradiated did not induce dendritic cells to secrete similar cytokines and chemokines or in magnitude. The *V*. *cholerae* cells in both the heat and chemically inactivated as well as solar irradiated cultures were no longer viable. However, the solar irradiated *V*. *cholerae* cultures induced the secretion of a wider variety of cytokines and chemokines as well as in magnitude when compared to the heat and chemically inactivated cultures. A combination of heat and chemicals, specifically formalin, has been one of the ways the cholera vaccine has and still is produced [59]. However, this mode of preparation does not induce dendritic cells to secrete diverse cytokines and chemokine as those observed when solar irradiated cultures are used. It is possible that the strains used for the preparation of the cholera vaccine are more immunogenic as opposed to those used in this study. We observed that the magnitude of the cytokines and chemokines secreted by the dendritic cells in similar treatments was strain specific. The identification of an ideal strain of *V*. *cholerae* may be critical when considering the use of SODIS treatment of water as a method of vaccine production.

In conclusion, the consumption of SODIS water containing waterborne pathogens may induce a desired immune response in consumers. Our results showed that solar irradiated cultures of *V*. *cholerae* induced the dendritic cells to secrete a diverse array of cytokines and chemokines than that observed when heat and chemically inactivated as well as PBS cultures were used. Furthermore, the concentration of the cytokines and chemokines produced by the dendritic cells in response to the solar irradiated cultures of *V*. *cholerae* was significantly higher than that observed in the untreated dendritic cells. However, the concentration of the cytokines and chemokines was not as high as that observed when LPS and non-solar irradiated cultures of *V*. *cholerae* in water were used to stimulate the dendritic cells. The profile of cytokines and chemokines secreted by the dendritic cells in response to solar irradiated cells would most likely elicit a Th2 immune response. However, mixed leukocyte assays as well as in vivo studies are required to show the exact phenotype of T-cells that are activated by these dendritic cells.
